# Reported healthcare-seeking of loiasis patients and estimation of the associated monetary burden in Gabon: Data from a cross-sectional survey

**DOI:** 10.1371/journal.pntd.0012389

**Published:** 2024-08-19

**Authors:** Luzia Veletzky, Veronika Schlicker, Jennifer Hergeth, Daniel R. Stelzl, Rella Zoleko Manego, Ghyslain Mombo-Ngoma, Kirsten Alexandra Eberhardt, Matthew B. B. McCall, Ayôla A. Adegnika, Bertrand Lell, Benjamin Mordmüller, Scherif Adegnika, Michael Ramharter, Christine Budke

**Affiliations:** 1 Department of Medicine I, Division of Infectious Diseases and Tropical Medicine, Medical University of Vienna, Vienna, Austria; 2 Centre de Recherches Médicales de Lambaréné, Lambaréné, Gabon; 3 Department of Tropical Medicine, Bernhard Nocht Institute for Tropical Medicine & I. Dep. of Medicine, University Medical Center Hamburg-Eppendorf, Hamburg, Germany; 4 German Center for infection Research, Partner Site Hamburg-Lübeck-Borstel-Riems, Germany; 5 Department of Urology, University Medical Center Hamburg-Eppendorf, Hamburg, Germany; 6 Department of Implementation Research, Bernhard Nocht Institute for Tropical Medicine & I. Dep. of Medicine, University Medical Center Hamburg-Eppendorf, Hamburg, Germany; 7 Radboud University Medical Center, Department of Medical Microbiology, Nijmegen, The Netherlands; 8 Institute of Tropical Medicine, University of Tübingen, Tübingen, Germany; 9 German Center for Infection Research (DZIF), partner site Tübingen, Tübingen, Germany; 10 Department of Veterinary Integrative Biosciences, College of Veterinary Medicine and Biomedical Sciences, Texas A&M University, College Station, Texas, United States of America; Federal University of Agriculture Abeokuta, NIGERIA

## Abstract

**Background:**

Loiasis is a disease of relevance in endemic populations and there has been advocacy for its inclusion on the World Health Organization’s neglected tropical diseases list. As loiasis-related healthcare-seeking behaviors and related costs are unknown, we aimed to evaluate these aspects in a population residing in an endemic region in Gabon.

**Methods:**

Data were collected during a community-based, cross-sectional study assessing the disease burden due to loiasis. Diagnostics for microfilaremia were performed and a history of eyeworm was obtained. In addition, a standardized questionnaire about type of healthcare resources and frequency of use, as well as respective associated costs was administered to each participant. Loiasis related healthcare-seeking behaviors were evaluated, and the associated monetary burden was estimated as a secondary outcome of the study.

**Findings:**

Individuals diagnosed with loiasis more frequently reported any healthcare-seeking (OR 1.52 (95%CI: 1.21–1.91)), self-medicating (OR 1.62 (1.26–2.08)), inability to work (OR 1.86 (1.47–2.35)), and consulting with traditional healers (logOdds 1.03 (0.52–1.53)), compared to loiasis negative individuals. The most frequently reported treatment for the eyeworm was traditional herbs. The estimated healthcare associated costs, per positive individual, was US-$ 58 (95% CI: 21–101) per year, which would correspond to 3.5% of the reported mean household income. Extrapolation to the rural population of Gabon (n = 204,000), resulted in an annual monetary burden estimate of US-$ 3,206,000 (1,150,000–5,577,000).

**Interpretation:**

Loiasis patients have demonstrated healthcare needs, often consulted traditional healers, and used traditional treatments for disease specific symptoms. Further, loiasis seems to be associated with substantial direct and indirect costs for individuals and thus may cause a relevant economic burden for endemic populations and economies of affected countries.

## Author summary

Loiasis has long been considered a negligible infection, however disease perception is changing as more data on significant disease-associated morbidity and mortality are being published. Still, many important aspects of the disease, such as the socioeconomic impact, are unknown. This study was part of a larger project on loiasis associated disease burden and aimed to evaluate healthcare seeking behaviors and estimate associated costs in individuals with loiasis living in a highly endemic region. We found that loiasis is associated with healthcare consultation, self-medicating, and sick leave. Self-medicating for treatment of disease specific symptoms was frequent. Traditional treatments and pain medication were most frequently used. Based on these data, we estimated loiasis related direct healthcare costs and indirect costs due to sickness, resulting in a monetary burden of US-$ 58 (95% CI: 21–101) per loiasis positive individual per year. Extrapolating these numbers to the rural population of Gabon (n = 204,000) would correspond to a monetary burden of US-$ 3,206,000 (95% CI: 1,150,000–5,577,000). Thus, loiasis is not only associated with morbidity and mortality but also with healthcare-seeking and may cause a relevant socioeconomic burden on affected populations. Therefore, considering loiasis a negligible disease is not appropriate anymore.

## Introduction

Loiasis, caused by the filarial worm *Loa loa*, has traditionally been considered as a benign infection in endemic areas. In the past, studies have focused on problems arising from co-endemicity with other filarial infections rather than on the impact of *Loa loa* itself. Importantly, the World Health Organization (WHO) has yet to categorize loiasis as a neglected tropical disease (NTD). However, the disease’s impact on endemic populations is increasingly being recognized and important associated mortality and morbidity, including major organ damage, have been described.[1–9] Consequently, it has been argued by the scientific community that loiasis should be acknowledged as an NTD.[10–12] Loiasis-related symptoms have been described as a major reason for consultations in highly endemic areas and use of traditional treatments against disease specific symptoms have been reported.[3,4,13] However, loiasis-related healthcare-seeking behaviors as well as the associated monetary burden at the individual and population levels have never been quantified. Insight into its economic burden is necessary to fully understand the impact of loiasis on affected populations, as is understanding healthcare-seeking behaviors for developing adequate future public health programs against the disease. This study aimed to evaluate the healthcare-seeking behaviors, including choice of healthcare, use of self-medication, and sick-leave associated with loiasis in an endemic population in Gabon. Based on these data, first estimates on the possible direct and indirect healthcare-associated costs and the monetary burden for individual loiasis patients and for the rural population of Gabon were generated.

## Methods

### Ethics statement

Ethical approval for the study was obtained from the Comité d’Ethique Institutionnel du Centre de Recherches Médicales de Lambaréné; CEI-011/2017. Written informed consent was obtained from all participants or by a parent or guardian in case of minors, before any study-related procedures were carried out.

### Data collection

Data for this analysis were gathered during a study aiming to evaluate the loiasis associated disease burden. Detailed methods on data collection were previously described.[2,7] In short, a community-based cross-sectional survey was conducted in 2017 and 2018 in rural villages in Gabon. Sampling within the villages was done per convenience screening. Residents older than 1 year of age were invited to participate in the study. A standardized questionnaire was administered by trained study personnel to collect baseline demographic information (see Table B in S1 Table). History of eyeworm was captured using the RAPLOA questionnaire.[2,14] Information on Calabar swelling, arthralgia, severe headaches, fatigue, paresthesia and transient paralysis of the extremities was also collected.[2] Calabar swelling and eyeworm history were queried for the last year and for the participant’s lifetime. Occurrence of all other loiasis associated symptoms was queried for the previous three months and participants were asked if they had sought healthcare for any of the symptoms (Table A in S1 Table). In the event of an affirmative response, the type of sought healthcare, including formal healthcare like hospitals and local healthcare centers, or informal healthcare, like traditional healers, was noted. Further, participants were asked if they used transport to get to the healthcare facility and if they took medication to soothe their symptoms, along with the respective associated costs. In case of reported self-medicating, the type of medication taken was asked. If provided by the interviewee, substance names were noted. Individuals who reported a history of eyeworm were asked how they had treated the eyeworm. Finally, individuals were asked if the reported symptoms had kept them from work during the previous year and for how many days. Initially participants were asked about their monthly household income, but the question was subsequently left out as it caused discomfort in the interviewees. Data were collected on paper-based forms and transferred to an electronic database. For quality control, 10% of the data were double-checked and since less than 5% of the data entries were incorrect the data entry quality was considered sufficient. A peripheral venous blood sample was collected between 10 am and 3 pm for diurnal microfilaremia detection. Microfilaremia detection was done using a total of 20ul blood on thick smears or, in case of smear negativity, by leukoconcentration of 1ml blood, as described previously.[2,7]

### Statistical analysis

**Fig 1:**
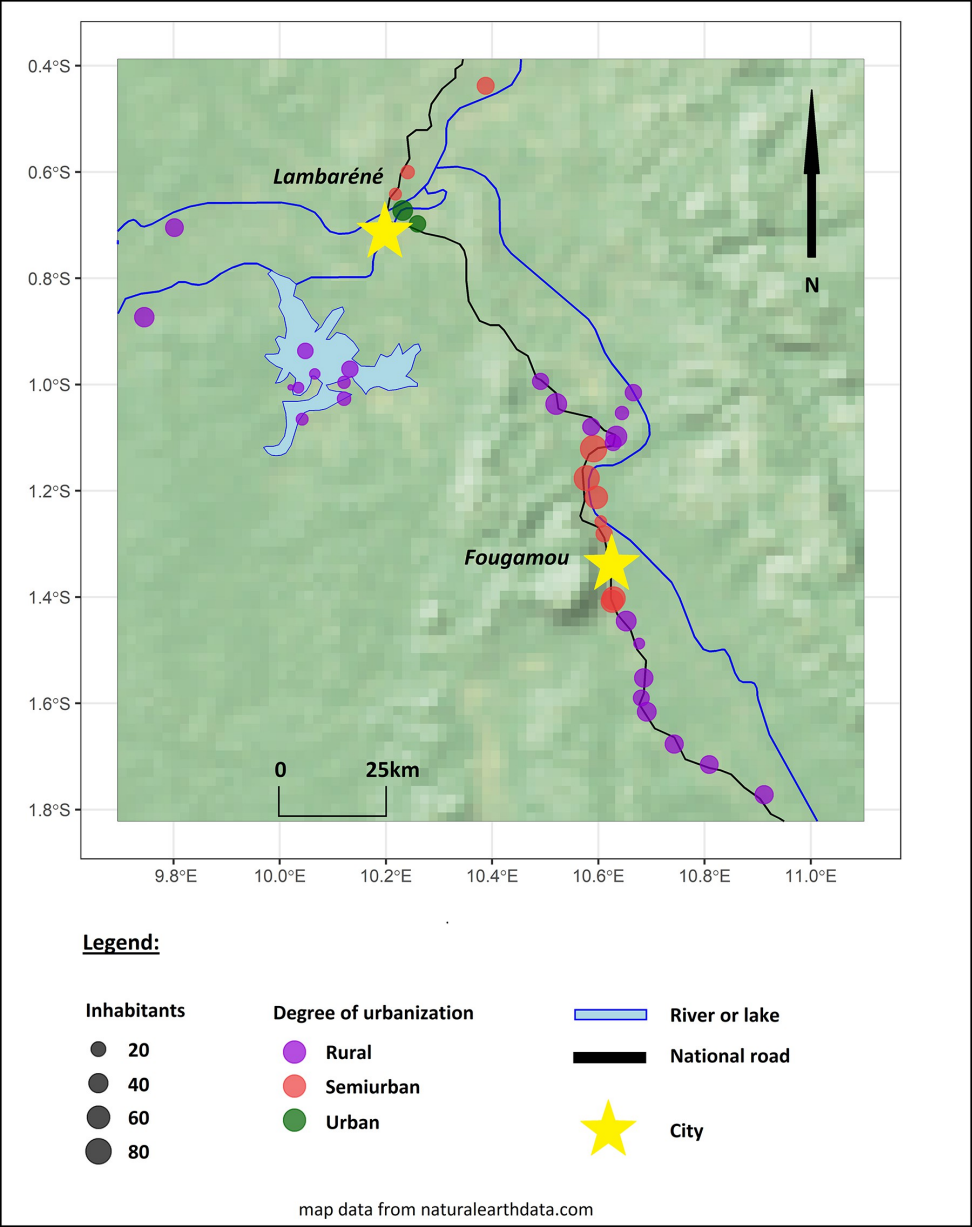
Study area and study population. Villages are defined as urban, semiurban and rural depending on proximity to Lambaréné or Fougamou and their population density.[2] Fig 1 was created in R using raster and vector map data from naturalearthdata.com (Version 4.0.5, R Foundation for Statistical Computing, Vienna, Austria).[18].

Study participants were grouped into loiasis positive and negative individuals. Loiasis positivity was defined as detectable microfilaremia and/or positive history of eyeworm according to the RAPLOA questionnaire. Participants were grouped into three age categories (<15, 15–59 and > = 60 years). Descriptive statistics were used to present frequencies of healthcare-seeking, type of healthcare sought, use of transportation, self-medicating, and sick leave. Associations between loiasis positivity, presence of microfilaremia and eyeworm positivity with healthcare-seeking variables were assessed by univariate analysis using the chi-2 test and calculation of odds ratios with 95% confidence intervals. Associations with categorical variables with more than two categories were calculated using multinomial logit regression analysis (logOdds). Continuous variables were expressed as medians and interquartile range (IQR). Sex, age group, proximity to road and degree of urbanization of villages were considered possible confounding factors and adjustment was done using logistic regressions for multivariable analysis. Relative risk ratios were obtained from the multinominal logit models. Comparison of median costs was done using Mann-Whitney-U test or Kruskal-wallis test, as appropriate. Two-sided p-values were presented, and an α of <0.05 was determined as significant. Data analysis was conducted in STATA 16 (StataCorp, College Station, TX). Fig 1 was created using open street map in R.[15]

### Monetary burden estimate

To calculate the monetary burden caused by loiasis, clinical and epidemiological data from the current study and information provided by the national health insurance scheme of Gabon (Caisse Nationale d’Assurance Maladie et de Garantie Sociale, CNAMGS), were used. Only data from the loiasis positive population were considered for the calculation. Frequencies of consultations, use of transportation, self-treatment and sick leave were directly extracted from the study data as were costs of respective items if provided by study participants. For costs not queried during the study, such as consultation fees, costs for laboratory analysis, or eyeworm removal, public price information provided by CNAMGS were used. Additional costs, including food for the day of travel, were estimated based on local prices of daytime meals. Table 1 provides an overview on items included in the calculation. The proportional contribution of each item (consultation, sick leave, etc.) was simulated for a loiasis case for a year. Uniform, normal and gamma distributions were applied to frequencies and item costs based on perceived uncertainty and visualization of the data to account for interindividual variations.

**Table 1:** Items applied to estimate the monetary burden of loiasis and their respective frequencies and distributions. °US-$ = US-Dollar, *number of individuals providing information in that cost, **in days per year, ^§^pricing of procedures in public health facilities provided by the Caisse Nationale d’Assurance Maladie et de Garantie Sociale (CNAMGS).

Items (direct costs)	N*	Median US-$°	IQR US-$°	Min-max US-$°	Costs distribution	Applied frequency	Frequency distribution	Data source
**Consultation costs**	NA	15	NA	NA	Uniform	53.3%	Uniform	CNAMGS^§^
**Medication costs**	244	4	2.5–11.66	NA	Gamma	69.0%	NA	Survey
**Laboratory costs**	NA	0.4	NA	NA	Normal	1.0%	NA	CNAMGS^§^
**Eyeworm removal costs**	NA	20	NA	NA	Normal	0.6%	NA	CNAMGS^§^
**Transport costs**	200	7	3–20	0.2–133	Gamma	61.9%	Gamma	Survey
**Extra food at day of consultation**	NA	2	NA	0.8–3	Uniform	0.5%	NA	Estimate
**Items (indirect costs)**	N*	**Median days**	**IQR days**	**Min-max days**	**Days distribution**	**Applied frequency**	**NA**	**Data source**
Sick leave per year**	289	7	3–14	1–365	Uniform	56.9%		Survey
	**N**	**Median US-$°**	**IQR US-$°**	**Min-max US-$°**	**Income distribution**	**NA**	**NA**	**Data source**
**Monthly income loiasis positives**	8	133	75–167	8–333	Normal	NA	NA	Survey

Items were grouped into direct and indirect costs and finally summed up to obtain the total costs. Direct costs were defined as the costs that are directly paid by the patient, including consultation fees, medication, blood analysis, eyeworm removal, transportation to the healthcare center and food for the day of consultation. Indirect costs included loss of income due to inability to work, with estimates based on the median number of reported sick leave days due to loiasis-related symptoms and assuming the median daily income as reported by study participants. Monte Carlo simulations with 10,000 iterations for each simulation were run using @Risk software (Lumivero, Denver, Colorado). Using individual estimates and regional loiasis prevalence data, costs were extrapolated to the total rural population of Gabon in 2017 of about 204,000.[2] Region and country level population numbers were obtained from the World Population Prospects for 2017 and from the 2013 Gabonese census.[16,17] Monetary burden was first calculated in Franc de la Coopération Financière en Afrique Centrale (CFA) and then converted to US-Dollar (US- $), using the exchange rate of 1 to 600.1 on the 31^st^ March 2023.

## Results

### Study population

A detailed description of the study population has been previously provided.[2,7] A total of 1,232 participants from 38 villages situated near the cities of Lambaréné and Fougamou took part in the cross-sectional survey (Fig 1 and Tables B and C in S1 Table).[2,7] Most participating villages were situated along the main road (55.3%, 21/38) and were defined as rural (68.4%, 26/38), comprising 62.1% and 59.3% of participants (765 and 730/1,232), respectively. Overall, 50.8% (626/1,232) of participants were found to be loiasis positive, including microfilaremic and/or eyeworm positive individuals. A univariate analysis was carried out to assess the effect of the degree of urbanization and distance to the main road on loiasis prevalence. Distance to the main road was significantly associated with microfilaremia (p = 0.003, Table C2 in S1 Table), but not with overall loiasis, nor with history of eyeworm. Individuals living in urbanized villages had significantly lower odds for being loiasis positive (p = 0.016, Table C1 in S1 Table), and for having a positive history of eyeworm (p = 0.038, Table C1 in S1 Table). There was no difference in frequency of microfilaremia between the populations of urban, semiurban and rural villages (Table C1 in S1 Table).

### Healthcare-seeking behaviors

Of all participants, 48.3% (582/1,206) sought healthcare for clinical manifestations that have been previously associated with *Loa loa* infection (Table 2). Individuals over 15 years of age reported consultations more often than younger participants (p = 0.022), and females slightly more often than males (p = 0.085, Table D1 in S1 Table). The type of consultation was reported by 98.5% (573/582) of healthcare-seeking participants, of which 77.0% went to a formal and 16.6% to an informal healthcare provider, while 6.4% went to both (Table 2). There was no statistically significant difference in healthcare type by sex or age group (Table D2 in S1 Table). Individuals living in proximity to the main road had increased odds for consulting a healthcare provider (p = 0.002, Table E1 in S1 Table) compared to individuals living farther from the main road, but the type of healthcare sought was similar. While a greater proportion of individuals from rural and semiurban populations consulted with informal healthcare providers compared to those from the urban population, this difference was not statistically significant nor was there a notable difference in consultation frequency by level of urbanization (Table E2 in S1 Table).

**Table 2:** Reported healthcare-seeking and use of related items by loiasis status, eyeworm and microfilaria positivity. % show column percentages, *Adjusted for age group and proximity to main road, **adjusted for age group, proximity to road and urbanization,° adjusted for sex and age group. ^§^Log odds. ^†^ P value of log odds. Loiasis positivity was defined as detectable microfilaremia and/or positive history of eyeworm according to the RAPLOA questionnaire. Microfilaremia positivity was defined as detectable microfilaremia, irrespective of eyeworm status. Eyeworm positivity was defined as a positive RAPLOA questionnaire, irrespective of microfilaremia status.

	Overall (%)	Loiasis + (%)	Loiasis–(%)	p-value (Chi-2)	OR (95% CI)	Adj. p value	Adj. OR (95% CI)	Eyeworm + (%)	Eyeworm–(%)	p-value (Chi-2)	OR (95% CI)	Adj. p value	Adj. OR (95% CI)	Micro- filaria + (%)	Micro-filaria–(%)	p-value (Chi-2)
**Consultation (1,206 respondents, 97.9% of all participants)**																
No	624 (51.7%)	289 (46.7%)	335 (57.1%)					234 (45.2%)	390 (56.7%)					146 (49.8%)	478 (52.4%)	
Yes	582 (48.3%)	330 (53.3%)	252 (42.9%)	<0.001	1.52 (1.21–1.91)	0.003*	1.43 (1.12–1.81)*	284 (54.8%)	298 (43.3%)	<0.001	1.59 (1.26–2.00)	0.001*	1.51 (1.19–1.91)*	147 (50.2%)	435 (47.6%)	0.452
**Healthcare type (573 respondents, 98.5% of all participants)**																
Formal	441 (77.0%)	233 (71.3%)	208 (84.6%)		1.03 (0.52–1.53) ^§^	<0.001^†^		194 (68.6%)	247 (85.2%)		1.17 (0.68–1.65)^§^	<0.001^†^		103 (71.0%)	338 (79.0%)	
Informal	95 (16.6%)	72 (22.0%)	23 (9.3%)					68 (24.0%)	27 (9.3%)					29 (20.0%)	66 (15.4%)	
Both	37 (6.4%)	22 (6.7%)	15 (6.1%)	<0.001				21 (7.4%)	16 (5.5%)	<0.001				13 (9.0%)	24 (5.6%)	0.126
**Transportation used (550 respondents, 94.5% of all participants)**																
No	179 (32.5%)	106 (34.3%)	73 (30.3%)					90 (34.1%)	89 (31.1%)					45 (32.6%)	134 (32.5%)	
Yes	371 (67.5%)	203 (65.7%)	168 (69.7%)	0.319	NA	NA	NA	174 (65.9%)	197 (68.9%)	0.457	NA	NA	NA	93 (67.4%)	278 (67.5%)	0.985
**Self-medicating (1,082 respondents, 87.8% of all participants)**																
No	395 (36.5%)	171 (31.0%)	224 (42.2%)					135 (29.2%)	260 (41.9%)					90 (34.9%)	305 (37.0%)	
Yes	687 (63.5%)	380 (69.0%)	307 (57.8%)	<0.001	1.62 (1.26–2.08)	0.003**	1.49 (1.14–1.94)**	327 (70.8%)	360 (58.1%)	<0.001	1.75 (1.35–2.26)	<0.001**	1.66 (1.27–2.16)**	168 (65.1%)	519 (63.0%)	0.535
**Sick leave (1,139 respondents, 92.5% of all participants)**																
No	577 (50.7%)	249 (43.1%)	328 (58.5%)					189 (39.0%)	388 (59.2%)					131 (48.2%)	446 (51.4%)	
Yes	562 (49.3%)	329 (56.9%)	233 (41.5%)	<0.001	1.86 (1.47–2.35)	<0.001°	2.04 (1.59–2.62°	295 (61.0%)	267 (40.8%)	<0.001	2.27 (1.78–2.88)	<0.001°	2.38 (1.86–3.05°	141 (51.8%)	421 (48.6%)	0.345

Loiasis positivity was associated with a higher frequency of consultations (p<0.001), including when adjusted for age group and proximity to a major road (p = 0.003). Loiasis positive individuals consulted different healthcare types than loiasis negatives (p<0.001). Consultation of informal health was reported by 22.0% of loiasis positives but only by 9.3% of loiasis negatives. The log odds of seeking informal compared to formal consultation increased by 1.03 (0.52–1.53) in loiasis positives compared to negatives (p<0.001). For individuals seeking informal consultation the relative risk ratio for being loiasis positive was 2.79 (1.69–4.63) compared to individuals seeking formal advice (p<0.001). Eyeworm positive individuals reported a higher frequency of consultation (p<0.001), consulted different healthcare types than negatives (p<0.001), namely informal healthcare compared to eye worm negatives (24.0% and 9.3%). The log odds of seeking informal compared to formal healthcare increased by 1.17 (0.68–1.65) in eyeworm positives (p<0.001). Microfilaremic and amicrofilaremic individuals reported a similar consultation frequency (p = 0.452) and consultation of healthcare types (p = 0.126). For details of comparison of loiasis positives and negatives see Table 2.

### Healthcare related items

**Fig 2:**
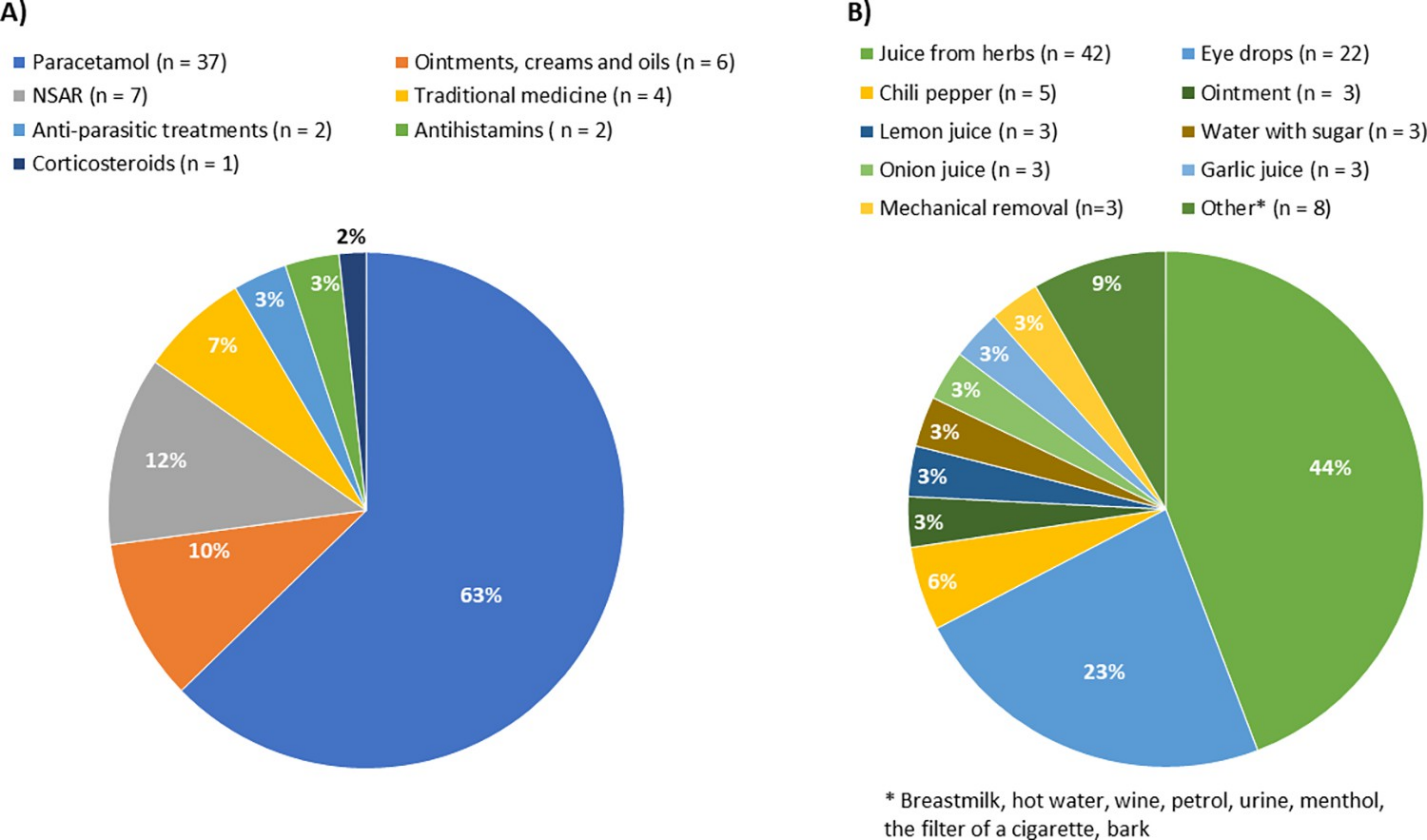
**Reported drugs used to relieve loiasis related symptoms (A) and treatments for eyeworm (B).** Paracetamol, followed by various non-steroidal antirheumatic drugs (NSAR), traditional treatment and topically applied substances were named most frequently. Eyeworm treatments comprised a variety of substances, including frequently named liquids such as herbal juices, eyedrops, but also chili, and various other substances*.

Of the individuals reporting healthcare consultations, 67.5% (371/550) used a form of transportation to reach the healthcare facility, while the remainder walked. There was no notable difference in use of transportation by sex, age group, loiasis positivity or distance to a main road (Tables D1 and E1 in S1 Table). Individuals living in rural and semiurban villages more frequently used transportation, compared to individuals living in urbanized villages (p = 0.015, Table E2 in S1 Table). Of all study participants, 63.5% (687/1,082) indicated that they did self-medicate for at least one of the queried symptoms (Table 2). While female and male participants reported a similar frequency of self-medicating, older age was associated with a higher frequency of self-medicating (p<0.001, Table D1 in S1 Table). Self-medicating was more frequently reported by participants living in rural and semiurban compared to individuals living in urbanized villages (p = 0.008, Table E2 in S1 Table), by individuals living in proximity to the main road compared to individuals living far from the main road (p<0.001, Table E1 in S1 Table) and by loiasis positives compared to loiasis negatives (p<0.001, Table 2). This difference remained after adjustment for sex, age, proximity to road and degree of urbanization (p = 0.003). Eyeworm positivity was associated with increased self-medicating (p<0.001) but microfilaremia was not. Use of “analgesics”, “antibiotics”, “others” and “a combination of drug types” was reported by 47.1% (170/361), 1.9% (7/361), 37.7% (136/361) and 13.3% (48/361) of loiasis positives who reported self-medicating and the used drug type, respectively. Specific names or active ingredients of medications were provided 59 times by loiasis positive individuals (Fig 2A).

Out of 520 eyeworm positive individuals, 72 reported the use of specific treatments against the eyeworm (Fig 2B). As some individuals reported the use of several treatments, 92 substances and three mechanical removals were noted. One individual indicated that this was done by their grandmother but did not specify how the worm was removed. Two participants reported that the worm had been removed using a needle but did not specify who performed the procedure.

Of the study population, 49.3% (562/1,139) reported that during the previous year they had days where they were unable to work, go to school or perform their household tasks due to the reported symptoms (Table 2). Individuals from the working age group (15 to 59 years of age) reported this sick leave most frequently (p<0.001, Table D1 in S1 Table), and female participants reported sick leave more frequently than males (p = 0.007, Table D1 in S1 Table). The degree of urbanization and proximity to a main road had no impact on reported frequency of sick leave (Tables E1 and E2 in S1 Table). Loiasis positivity was associated with a higher frequency of sick leave (p<0.001), also when adjusted for sex and age group, as was eyeworm positivity (p<0.001), while microfilaremia was not (Table 2).

### Monetary burden

**Fig 3:**
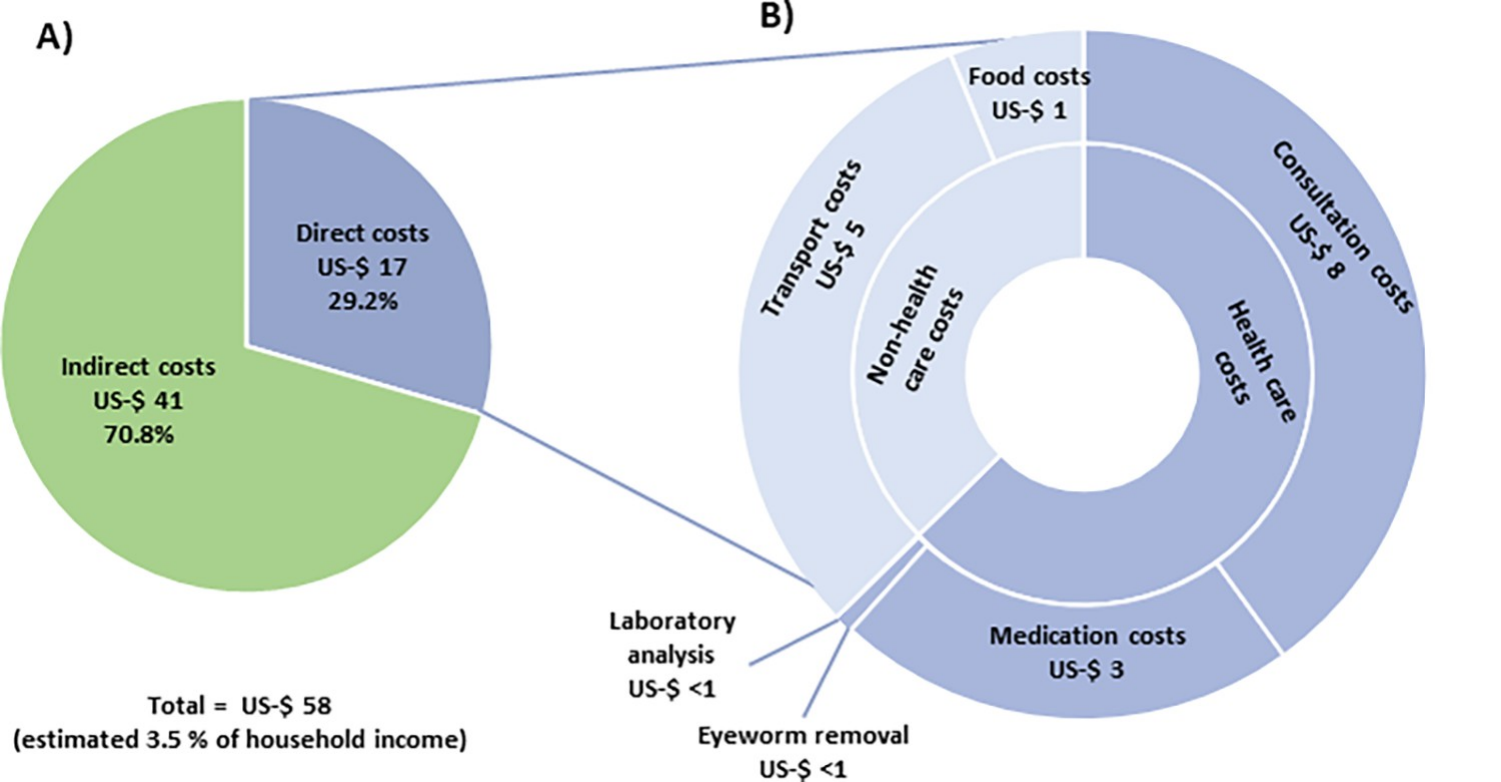
**Estimated loiasis associated costs:** Median total costs, including direct and indirect costs for loiasis positive individuals (A), detailed costs of different items and their proportion in direct costs (B).

Data on monthly household income were available from 21 participants, including 13 (61.9%) loiasis negative participants and 8 (38.1%) loiasis positive participants. Loiasis negative individuals reported a median monthly household income of US-$ 167 per month, while loiasis positive individuals reported a median of US-$ 133, (Mann-Whitney-U test p = 0.435). Males reported a median monthly household income of US-$ 167 and female participants a median monthly household income of US-$ 100, (Mann-Whitney-U test p = 0.025). Individuals aged 60 years or older reported a median monthly household income of US-$ 75, while younger adults or families with children reported a median of US-$ 167, (Kruskal-wallis p = 0.165). Transportation costs to healthcare were provided by 200 loiasis positive individuals, with a median of US-$ 7. Medication costs were provided by 244 individuals, with a median of US-$ 4. Sick leave during the previous year due to loiasis related symptoms was reported by 329 loiasis positive individuals. Of those, 289 provided information on the duration and took a median of 7 days per year (IQR: 3–14) (Table 1). The estimated median cost for a loiasis positive individual was US-$ 58 (95% CI: 21–101) per year, including US-$ 17 (14–22) for direct costs and US-$ 41 (7–79) for indirect costs (Fig 3A and 3B). Healthcare costs represented 64.7% (US-$ 11) of direct costs, while non-healthcare costs contributed the remaining 35.3% (US-$ 6). Extrapolation to the entire loiasis positive rural population of Gabon resulted in US-$ 936,000 (773,000–1,194,000) of direct and US-$ 2,270,000 (377,000–4,382,000) of indirect costs, causing a total of US-$ 3,206,000 (1,150,000–5,577,000) costs for a population of about 204,000 inhabitants (see Tables F and G in S1 Table for detailed results in CFA and US-$).

## Discussion

As more data on loiasis-related mortality and morbidity are becoming available, the call for loiasis to be recognized as a NTD by the WHO grows louder.[11,12] At the same time, it is evident that there is still much to learn about the disease and its impact on affected populations. Next to the associated health consequences, the socioeconomic burden is a major component of a comprehensive understanding of the disease. Here we present the first systematically gathered data on loiasis-related healthcare-seeking behaviors and based on these data, a first estimate of the possible associated monetary burden. We show that loiasis is associated with healthcare-seeking, self-medicating, and inability to work, probably leading to substantial direct and indirect costs for affected individuals.

We estimated that loiasis positivity could be associated with a median monetary burden of US-$ 58 per year for the individual, including US-$ 17 of direct and US-$ 41 of indirect costs. Based on the reported incomes this would correspond to 3.5% of the median total reported yearly household income. Considering similar disease prevalence in the entire rural population of Gabon we extrapolated the data and estimated an absolute annual monetary burden of US-$ 3,206,900 in this population due to loiasis. While there are no previous data on the monetary burden due to loiasis, one may compare data on NTDs from the region. One study on cysticercosis in Cameroon estimated that the monetary burden during a year, including full treatments and loss of income, was about US-$ 210 per case.[19] Another study from Nigeria evaluated the monetary burden of onchocerciasis and estimated monthly treatment costs of US-$ 14 and monthly loss of income of US-$ 16.^19^ A more recent study estimated treatment costs of about US-$ 73 per affected individual, however there was no timeframe provided.[20,21] Considering that the presented data for loiasis is still incomplete, the associated monetary burden of loiasis may, therefore, be of a similar magnitude to that of WHO-recognized NTDs.

Microfilaremia is increasingly recognized as a factor for serious disease outcomes in loiasis, including increased mortality and organ damage.[1,6,8,9] In our study, however, microfilaremic individuals did not report more healthcare-consultation due to loiasis associated symptoms, compared to loiasis negatives, and less than eyeworm positives. This is in line with our previous finding that microfilaremic individuals experience fewer subjective symptoms compared to eyeworm positive individuals.[7,22] It may be speculated that the subjective well-being or lack of specific symptoms leads to less healthcare-seeking in these individuals at highest risk for severe disease outcomes. Though the severe disease outcomes are estimated to be rare, they may cause a substantial burden on individuals and at the household-level. While this work did not aim to evaluate the effects of these major health complications, which is an important limitation, it is thus even more compelling that despite their omission, the estimated monetary burden was still considerable high.

Similar to other NTDs, loiasis predominately impacts rural and poorer populations in Central Africa and thereby stands in the way of the United Nations Sustainable Development Goals (SDGs) by not only causing disease but also increasing inequalities between, and importantly within populations.[23,24] This divide is likely driven not only by differences in disease prevalence, but also by availability of and access to healthcare. We, therefore, specifically evaluated possible effects of urbanization and proximity to a main road on healthcare-seeking and related items. Individuals living in proximity to the main road reported seeking consultations more often and higher use of self-medication, which might indicate better access to both. Individuals living in rural villages, in contrast, reported use of transportation to healthcare facilities more often, supporting the hypothesis that individuals in rural areas have added barriers to access healthcare and thus additional direct costs.[25] Future studies should address these differences and look at specific needs of loiasis patients in rural and (semi-)urban populations in more detail.

Loiasis positive individuals tended to consult traditional healers more often for relief of their symptoms, which was even more pronounced in individuals with eyeworm positive loiasis. On the other hand, the most frequently substance groups used for self-medicating, paracetamol and NSAIDs, are widely available, frequently used drugs. This may reflect that there are no medications specifically used for loiasis, and that those affected simply revert to available substances. In contrast to this, it is striking that for the eyeworm traditional therapies were named most frequently. This may indicate difficulties in access to formal healthcare for loiasis related symptoms, especially for the eyeworm. This is further in line with anecdotal reports from patients that their symptoms are not taken seriously by members of the formal healthcare system. The formal healthcare sector lacks treatment options for loiasis, as specific drugs may not be available, or disease related symptoms may not be recognized. On the contrary, traditional healers in highly endemic regions may be more aware of the disease, offering specific treatment options and psychological support for affected individuals. Notably, we did not inquire about healthcare seeking specifically due to the symptoms of eyeworm and Calabar swelling, which is a limitation and would certainly be of interest in future studies.

The main sources of income in this rural population of central Gabon are subsistence farming, hunting, and fishing.[26] Self-reported information on mean household income was available for only the first 21 participants since this question caused social discomfort to participants. While our data on household income are very limited, they are in the range of expected income in this population (personal communication with Dr Adegnika). Further, it appears that older and female participants had a lower reported household income, representing a known pattern in these marginalized groups. Further, study participants reported a lower median monthly household income than the estimated minimum wage in Gabon of US-$ 250 per month, indicating an already financially precarious situation.[27]

We included direct healthcare-related costs such as consultation fees and costs of drugs, as well as non-healthcare costs such as travel to healthcare. Non-healthcare related direct costs, however, may disproportionally affect loiasis patients due to their lower median household income compared to loiasis negatives. Importantly, indirect costs such as loss of income due to an inability to work must not be omitted. However, we did not differentiate between non-payment of salary and loss of income due to inability to work in subsistence farming or hunting. While the latter would comprise loss of income due to the inability to sell agricultural products or provide food for the individual or their family, the former would also result in loss of taxes for the state and impact the country’s economy. Both would be important factors to be addressed in more comprehensive, future studies.

Another important limitation is that some symptoms may have been incorrectly assigned to loiasis, resulting in reported healthcare-seeking by loiasis positive individuals that was due to other diseases. Further, due to questionnaire-based, retrospective data collection, symptom frequency, timeframe, and healthcare-seeking may have been influenced by recall bias. Additionally, healthcare seeking, self-medicating and sick leave frequency was overall quite high. All these factors may have led to an overestimation of costs. To account for these uncertainties and to limit the risk of overestimation, we included conservative estimates of the associated costs, applied distributions to the estimates and adjusted for confounding factors. Furthermore, it is important to recognize that in areas with high prevalence of multi-parasitism and limited healthcare, a clear disentanglement of impacts of different diseases is difficult. The data forming the basis of this study were collected in 2017–2018 and the monetary values may have changed since then. This may have caused a significant underestimation of current costs. Nevertheless, these data will hopefully pave the way for future, more detailed studies, using qualitative data and addressing the multifaceted aspects of loiasis disease.

## Conclusion

This is the first study to analyze healthcare-seeking and estimate associated costs for loiasis positive individuals living in a highly endemic area of central Africa. The presented data show substantial healthcare-seeking and associated monetary burden due to the disease, further supporting the call for the end of the neglect of loiasis and its appreciation as a clinically important disease that necessitates the development of adequate elimination and treatment strategies.

## Supporting information

S1 Table Table A. Healthcare related questionnaire used during the survey. * in CFA-Franc (Franc de la Coopération Financière en Afrique Centrale). Table B. Overview on baseline data including age, sex and loiasis infection states. Table C. (1) Overview on baseline data including age, sex and loiasis infection states by degree of urbanization. (2) Overview on baseline data including age, sex and loiasis infection states by proximity to the main road. Table D. 1) Overview on healthcare-seeking, transport, self-medicating and sick leave by sex and age. 2) Overview on healthcare type by sex and age. Table E. 1) Overview on healthcare-seeking, healthcare type, transport, self-medicating and sick leave by proximity to road (on the road vs. off the road) and 2) degree of urbanisation. Table F. Cost estimates in CFA. Table G. Cost estimates in USD.
(DOCX)

S1 Database Database of the study. (XLS)
