# Supplementary material for: Reported healthcare-seeking of loiasis patients and estimation of the associated monetary burden in Gabon: Data from a cross-sectional survey
Source: PLoS Negl Trop Dis. 2024 Aug 19;18(8):e0012389. doi: 10.1371/journal.pntd.0012389 (PMC11361742; doi:10.1371/journal.pntd.0012389)
Supplement: S1 Table — Table A. Healthcare related questionnaire used during the survey. * in CFA-Franc (Franc de la Coopération Financière en Afrique Centrale). Table B. Overview on baseline data including age, sex and loiasis infection states. Table C. (1) Overview on baseline data including age, sex and loiasis infection states by degree of urbanization. (2) Overview on baseline data including age, sex and loiasis infection states by proximity to the main road. Table D. 1) Overview on healthcare-seeking, transport, self-medicating and sick leave by sex and age. 2) Overview on healthcare type by sex and age. Table E. 1) Overview on healthcare-seeking, healthcare type, transport, self-medicating and sick leave by proximity to road (on the road vs. off the road) and 2) degree of urbanisation. Table F. Cost estimates in CFA. Table G. Cost estimates in USD. (DOCX) [file pntd.0012389.s001.docx]

**Supporting information**

**Table A. Healthcare related questionnaire used during the survey. * in CFA-Franc (Franc de la Coopération Financière en Afrique Centrale)**

| **Healthcare-seeking behavior specific questionnaire** | | | |
| --- | --- | --- | --- |
| Question | Possible answers | | |
| Did you experience <arthralgia, severe headaches, fatigue, paresthesia and transient paralysis of the extremities> during the previous three months? | Yes | No | - |
| Did you experience transient swellings of the wrists in the last year? | Yes | No |  |
| Did you ever experience transient swellings of the wrists? | Yes | No |  |
| Raploa questionnaire for the previous year and for life-time | Yes | No |  |
| Did you seek healthcare because of any of the above-mentioned symptoms? | Yes | No | - |
| If yes, which kind? | Traditional healer | Local healthcare center (HCC) | Hospital |
| Did you take transportation to go there? | Yes | No | - |
| If yes, how much did it cost? | Amount noted in CFA | | |
| Did you take medication to improve the symptoms? | Yes | No | - |
| If yes, which kind? | Painkillers | Antibiotics | Other |
| Name or substance provided by the patient. | Response noted | | |
| If yes, how much did it cost? | Amount noted in CFA | | |
| Did any of the symptoms keep you from doing your daily work? | Yes | No | - |
| If yes, how many days during the last year? | In days | | |
|  | | | |
| **Data available from subgroups** | | | |
| Did you use specific treatment against the eyeworm and if yes which one?* | Response noted | | |
| * was not systematically queried, responses noted by study personnel | | | |

**Table B. Overview on baseline data including age, sex and loiasis infection states.**

| **Total study cohort overview** | | |
| --- | --- | --- |
|  | **N total** | **Col%** |
| **Villages** | 38 | 100,00% |
|  |  |  |
| **Inhabitants** | 1232 | 100,00% |
|  |  |  |
| **Age** |  |  |
| **<15** | 157 | 12,70% |
| **15-59** | 751 | 61,00% |
| **>=60** | 324 | 26,30% |
| **Sex** |  |  |
| **Female** | 668 | 54,20% |
| **Male** | 564 | 45,80% |
| **Loiasis** |  |  |
| **Pos** | 626 | 50,80% |
| **Neg** | 606 | 49,20% |
| **Microfilaremia** |  |  |
| **Pos** | 298 | 24,20% |
| **Neg** | 934 | 75,80% |
| **Raploa** |  |  |
| **Pos** | 520 | 42,20% |
| **Neg** | 712 | 57,80% |
| **Col%= column percentages** | | |

**Table C. (1) Overview on baseline data including age, sex and loiasis infection states by degree of urbanization. (2) Overview on baseline data including age, sex and loiasis infection states by proximity to the main road.**

| **C1) By degree of urbanization** | | | | | | | |  |  |
| --- | --- | --- | --- | --- | --- | --- | --- | --- | --- |
|  | **N** | **Urban** | **Row%** | **Semiurban** | **Row%** | **Rural** | **Row%** |  |  |
| **Villages** | 38 | 2 | 5,30% | 10 | 26,32% | 26 | 68,42% |  |  |
| **Inhabitants** | 1232 | 68 | 5,50% | 434 | 35,23% | 730 | 59,25% |  |  |
| **Agegroup** | **N** | **Urban** | **Col%** | **Semiurban** | **Col%** | **Rural** | **Col%** | **p-value (Chi-2)** | **OR (95% CI)** |
| **<15** | 157 | 8 | 11,80% | 59 | 13,59% | 90 | 12,33% |  |  |
| **15-59** | 751 | 42 | 61,80% | 266 | 61,29% | 443 | 60,68% |  |  |
| **>=60** | 324 | 18 | 26,50% | 109 | 25,12% | 197 | 26,99% | 0.941 | NA |
| **Sex** | **N** | **Urban** | **Col%** | **Semiurban** | **Col%** | **Rural** | **Col%** | **p-value (Chi-2)** |  |
| **Female** | 668 | 40 | 58,82% | 227 | 52,30% | 401 | 54,93% |  |  |
| **Male** | 564 | 28 | 41,18% | 207 | 47,70% | 329 | 45,07% | 0.504 | NA |
|  | **N** | **Urban** | **Col%** | **Semiurban** | **Col%** | **Rural** | **Col%** | **p-value (Chi-2)** |  |
| **Loiasis negative** | 606 | 44 | 64,71% | 200 | 46,08% | 362 | 49,59% |  |  |
| **Loiasis positive** | 626 | 24 | 35,29% | 234 | 53,92% | 368 | 50,41% | 0.016 | 0.78 (0.64-0.96) |
|  | **N** | **Urban** | **Col%** | **Semiurban** | **Col%** | **Rural** | **Col%** | **p-value (Chi-2)** |  |
| **Amicrofilaremic** | 934 | 55 | 80,88% | 323 | 74,42% | 556 | 76,16% |  |  |
| **Microfilaremic** | 298 | 13 | 19,12% | 111 | 25,58% | 174 | 23,84% | 0.482 | NA |
|  | **N** | **Urban** | **Col%** | **Semiurban** | **Col%** | **Rural** | **Col%** | **p-value (Chi-2)** |  |
| **Raploa negative** | 712 | 48 | 70,59% | 237 | 54,61% | 427 | 58,49% |  |  |
| **Raploa positive** | 520 | 20 | 29,41% | 197 | 45,39% | 303 | 41,51% | 0.038 | 0.79 (0.65-0.97) |
| **Col% = column percentages, Row%= row percentages** | | | | | | | | | |

| **C2) By proximity to the main road** | | | | | | | |
| --- | --- | --- | --- | --- | --- | --- | --- |
|  | **N** | **On the main road** | **Row%** | **Off the main road** | **Row%** |  |  |
| **Villages** | 38 | 21 | 55,30% | 17 | 44,70% |  |  |
| **Inhabitants** | 1232 | 765 | 62,10% | 467 | 37,90% |  |  |
| **Agegroup** | **N** | **On the main road** | **Col%** | **Off the main road** | **Col%** | **p-value (Chi-2)** | **OR (95% CI)** |
| **<15** | 157 | 95 | 12,40% | 62 | 13,30% |  |  |
| **15-59** | 751 | 475 | 62,10% | 276 | 59,10% |  |  |
| **>=60** | 324 | 195 | 25,50% | 129 | 27,60% | 0.578 | NA |
| **Sex** | **N** | **On the main road** | **Col%** | **Off the main road** | **Col%** | **p-value (Chi-2)** | **OR (95% CI)** |
| **Female** | 668 | 421 | 55,00% | 247 | 52,90% |  |  |
| **Male** | 564 | 344 | 45,00% | 220 | 47,10% | 0.464 | NA |
|  | **N** | **On the main road** | **Col%** | **Off the main road** | **Col%** | **p-value (Chi-2)** | **OR (95% CI)** |
| **Loiasis negative** | 606 | 405 | 52,90% | 221 | 47,30% |  |  |
| **Loiasis positive** | 626 | 360 | 47,10% | 246 | 52,70% | 0.056 | 1.25 (0.99-1.58) |
|  | **N** | **On the main road** | **Col%** | **Off the main road** | **Col%** | **p-value (Chi-2)** | **OR (95% CI)** |
| **Amicrofilaremic** | 934 | 558 | 72,90% | 376 | 80,50% |  |  |
| **Microfilaremic** | 298 | 207 | 27,10% | 91 | 19,50% | 0.003 | 1.53 (1.16-2.03) |
|  | **N** | **On the main road** | **Col%** | **Off the main road** | **Col%** | **p-value (Chi-2)** | **OR (95% CI)** |
| **Raploa positive** | 712 | 336 | 43,90% | 184 | 39,40% |  |  |
| **Raploa negative** | 520 | 429 | 56,10% | 283 | 60,60% | 0.119 | NA |
| **Col% = column percentages, Row%= row percentages** | | | | | | | |

**Table D. 1) Overview on healthcare-seeking, transport, self-medicating and sick leave by sex and age. 2) Overview on healthcare type by sex and age.**

| **Table D1** | | | | | | | | |
| --- | --- | --- | --- | --- | --- | --- | --- | --- |
| **Consultation** | **N** | **Yes** | **Row%** | **No** | **Row%** | **p-value (Chi-2)** | **OR** | **CI** |
|  | 1206 | 582 | 48,26% | 624 | 51,74% |  |  |  |
| **Age** |  |  |  |  |  |  |  |  |
| **<15** | 148 | 56 | 37,84% | 92 | 62,16% |  |  |  |
| **15-59** | 744 | 366 | 49,19% | 378 | 50,81% |  |  |  |
| **>=60** | 314 | 160 | 50,96% | 154 | 49,04% | 0.022 |  |  |
|  |  |  |  |  |  |  |  |  |
| **Consultation** | **N** | **Yes** | **Row%** | **No** | **Row%** | **p-value (Chi-2)** | **OR** | **CI** |
|  | 1206 | 582 | 48,26% | 624 | 51,74% |  |  |  |
| **Female** | 653 | 330 | 50,54% | 323 | 49,46% |  |  |  |
| **Male** | 553 | 252 | 45,57% | 301 | 54,43% | 0.085 | NA | NA |
|  |  |  |  |  |  |  |  |  |
|  |  |  |  |  |  |  |  |  |
| **Transport used** | **N** | **Yes** | **Row%** | **No** | **Row%** | **p-value (Chi-2)** | **OR** | **CI** |
|  | 550 | 371 | 67,45% | 179 | 32,55% |  |  |  |
| **Age** |  |  |  |  |  |  |  |  |
| **<15** | 50 | 34 | 68,00% | 16 | 32,00% |  |  |  |
| **15-59** | 353 | 228 | 64,59% | 123 | 34,84% |  |  |  |
| **>=60** | 149 | 109 | 73,15% | 40 | 26,85% | 0.201 | NA | NA |
|  |  |  |  |  |  |  |  |  |
| **Transport used** | **N** | **Yes** | **Row%** | **No** | **Row%** | **p-value (Chi-2)** | **OR** | **CI** |
|  | 550 | 371 | 67,45% | 179 | 32,55% |  |  |  |
| **Female** | 319 | 212 | 66,46% | 105 | 32,92% |  |  |  |
| **Male** | 233 | 159 | 68,24% | 74 | 31,76% | 0.736 | NA | NA |
|  |  |  |  |  |  |  |  |  |
| **Selfmedication used** | **N** | **Yes** | **Row%** | **No** | **Row%** | **p-value (Chi-2)** | **OR** | **CI** |
|  | 1082 | 687 | 63,49% | 395 | 36,51% |  |  |  |
| **Age** |  |  |  |  |  |  |  |  |
| **<15** | 136 | 64 | 47,06% | 72 | 52,94% |  |  |  |
| **15-59** | 665 | 446 | 67,07% | 219 | 32,93% |  |  |  |
| **>=60** | 281 | 177 | 62,99% | 104 | 37,01% | <0.001 |  |  |
|  |  |  |  |  |  |  |  |  |
| **Selfmedication used** | **N** | **Yes** | **Row%** | **No** | **Row%** | **p-value (Chi-2)** | **OR** | **CI** |
|  | 1082 | 687 | 63,49% | 395 | 36,51% |  |  |  |
| **Female** | 599 | 392 | 65,44% | 207 | 34,56% |  |  |  |
| **Male** | 483 | 295 | 61,08% | 188 | 38,92% | 0.138 | NA | NA |
|  |  |  |  |  |  |  |  |  |
| **Impact on daily work** | **N** | **Yes** | **Row%** | **No** | **Row%** | **p-value (Chi-2)** | **OR** | **CI** |
|  | 1139 | 562 | 49,34% | 577 | 50,66% |  |  |  |
| **Age** |  |  |  |  |  |  |  |  |
| **<15** | 138 | 55 | 39,86% | 83 | 60,14% |  |  |  |
| **15-59** | 720 | 385 | 53,47% | 335 | 46,53% |  |  |  |
| **>=60** | 281 | 122 | 43,42% | 159 | 56,58% | <0.001 |  |  |
|  |  |  |  |  |  |  |  |  |
| **Impact on daily work** | **N** | **Yes** | **Row%** | **No** | **Row%** | **p-value (Chi-2)** | **OR** | **CI** |
|  | 1139 | 562 | 49,34% | 577 | 50,66% | 0.007 | 0.73 | 0.58-0.92 |
| **Female** | 615 | 326 | 53,01% | 289 | 46,99% | **adj.p-value*** | **aOR*** | **CI** |
| **Male** | 524 | 236 | 45,04% | 288 | 54,96% | 0.007 | 0.73 | 0.57-0.92 |
| *adjusted to sex and age. Row%= row percentages | | | | | | | | |
| **Table D2** | | | | | | | | |
| **Healthcare type** | **N** | **Formal** | **Row%** | **Informal** | **Row%** | **Both** | **Row%** | **p-value (Chi-2)** |
|  |  | 441 |  | 95 |  | 37 |  |  |
| **Age** | 573 |  |  |  |  |  |  |  |
| **<15** | 53 | 42 | 79,25% | 10 | 18,87% | 1 | 1,89% |  |
| **15-59** | 361 | 281 | 77,84% | 56 | 15,51% | 24 | 6,65% |  |
| **>=60** | 159 | 118 | 74,21% | 29 | 18,24% | 12 | 7,55% | 0.569 |
|  |  |  |  |  |  |  |  |  |
| **Healthcare type** | **N** | **Formal** | **Row%** | **Informal** | **Row%** | **Both** |  |  |
|  | 573 | 441 | 76,96% | 95 | 16,58% | 37 | 6,46% |  |
| **Female** | 327 | 258 | 78,90% | 52 | 15,90% | 17 | 5,20% |  |
| **Male** | 246 | 183 | 74,39% | 43 | 17,48% | 20 | 8,13% | 0.294 |
| **Row%= row percentages** | | | | | | | | |

**Table E. 1) Overview on healthcare-seeking, healthcare type, transport, self-medicating and sick leave by proximity to road (on the road vs. off the road) and 2) degree of urbanisation.**

| **Table E1** |  |  |  |  |  |  |  |  |  |
| --- | --- | --- | --- | --- | --- | --- | --- | --- | --- |
| **Consultation** | **N** | **Off** | **Col%** | **On** | **Col%** | **p-value (Chi-2)** | **OR (95% CI)** | **adj.p-value** |  |
|  | 1,206 | 459 |  | 747 |  |  |  |  |  |
| **No** | 624 | 263 | 57,30% | 361 | 48,33% |  |  |  |  |
| **Yes** | 582 | 196 | 42,70% | 386 | 51,67% | 0.002 | 1.44 (1.14-1.81) | 0.002 | 1.44 (1.14-1.82 |
|  |  |  |  |  |  |  |  |  |  |
| **Healthcare type** |  | **Off** | **Col%** | **On** | **Col%** | **p-value (Chi-2)** |  |  |  |
|  | 573 | 191 |  | 382 |  |  |  |  |  |
| **formal** | 441 | 150 | 78,53% | 291 | 76,18% |  |  |  |  |
| **informal** | 95 | 32 | 16,75% | 63 | 16,49% |  |  |  |  |
| **both** | 37 | 9 | 4,71% | 28 | 7,33% | 0.485 |  |  |  |
|  |  |  |  |  |  |  |  |  |  |
| **Transport used** |  | **Off** | **Col%** | **On** | **Col%** | **p-value (Chi-2)** |  |  |  |
|  | 550 | 189 |  | 361 |  |  |  |  |  |
| **No** | 179 | 63 | 33,33% | 116 | 32,13% |  |  |  |  |
| **Yes** | 371 | 126 | 66,67% | 245 | 67,87% | 0.775 |  |  |  |
|  |  |  |  |  |  |  |  |  |  |
| **Selfmedication used** | | **Off** | **Col%** | **On** | **Col%** | **p-value (Chi-2)** |  |  |  |
|  | 1,082 | 448 |  | 634 |  |  |  |  |  |
| **No** | 395 | 198 | 44,20% | 197 | 31,07% |  |  |  |  |
| **Yes** | 687 | 250 | 55,80% | 437 | 68,93% | <0.001 | 1.76 (1.37-2.26) |  | 1.77 (1.37-2.27) |
|  |  |  |  |  |  |  |  |  |  |
| **Impact on daily work** |  | **Off** | **Col%** | **On** | **Col%** | **p-value (Chi-2)** |  |  |  |
|  | 1,139 | 429 |  | 710 |  |  |  |  |  |
| **No** | 577 | 217 | 50,58% | 360 | 50,70% |  |  |  |  |
| **Yes** | 562 | 212 | 49,42% | 350 | 49,30% | 0.968 |  |  |  |

| **Table E2** |  |  |  |  |  |  |  |  |
| --- | --- | --- | --- | --- | --- | --- | --- | --- |
| **Consultation** | **N** | **Urban** | **Col%** | **Semiurban** | **Col%** | **Rural** | **Col%** | **p-value (Chi-2)** |
|  | 1,206 | 68 |  | 423 |  | 715 |  |  |
| **No** | 624 | 40 | 58,82% | 216 | 51,06% | 368 | 51,47% |  |
| **Yes** | 582 | 28 | 41,18% | 207 | 48,94% | 347 | 48,53% | 0.481 |
|  |  |  |  |  |  |  |  |  |
| **Healthcare type** |  | **Urban** | **Col%** | **Semiurban** | **Col%** | **Rural** | **Col%** | **p-value (Chi-2)** |
|  | 573 | 28 |  | 205 |  | 340 |  |  |
| **formal** | 441 | 22 | 78,57% | 162 | 79,02% | 257 | 75,59% |  |
| **informal** | 95 | 3 | 10,71% | 31 | 15,12% | 61 | 17,94% |  |
| **both** | 37 | 3 | 10,71% | 12 | 5,85% | 22 | 6,47% | 0.675 |
|  |  |  |  |  |  |  |  |  |
| **Transport used** |  | **Urban** | **Col%** | **Semiurban** | **Col%** | **Rural** | **Col%** | **p-value (Chi-2)** |
|  | 550 | 26 |  | 195 |  | 329 |  |  |
| **No** | 179 | 15 | 57,69% | 65 | 33,33% | 99 | 30,09% |  |
| **Yes** | 371 | 11 | 42,31% | 130 | 66,67% | 230 | 69,91% | 0.015 |
|  |  |  |  |  |  |  |  |  |
| **Selfmedication used** | | **Urban** | **Col%** | **Semiurban** | **Col%** | **Rural** | **Col%** | **p-value (Chi-2)** |
|  | 1,082 | 67 |  | 361 |  | 654 |  |  |
| **No** | 395 | 36 | 53,73% | 122 | 33,80% | 237 | 36,24% |  |
| **Yes** | 687 | 31 | 46,27% | 239 | 66,20% | 417 | 63,76% | 0.008 |
|  |  |  |  |  |  |  |  |  |
| **impact on daily work** |  | **Urban** | **Col%** | **Semiurban** | **Col%** | **Rural** | **Col%** | **p-value (Chi-2)** |
|  | 1,139 | 66 |  | 401 |  | 672 |  |  |
| **No** | 577 | 40 | 60,61% | 203 | 50,62% | 334 | 49,70% |  |
| **Yes** | 562 | 26 | 39,39% | 198 | 49,38% | 338 | 50,30% | 0.239 |

**Table F. Cost estimates in CFA.**

| **Per loiasis positive individual** | | | | | | | |
| --- | --- | --- | --- | --- | --- | --- | --- |
| **Cost Type** | **Item** | **Components** | **Estimated median costs** | **95% CI lower** | **95% CI upper** | **Min** | **Max** |
| **Direct costs** | **Health care costs** | **Consultation** | **4.664** | **3.749** | **5.578** | **2.841** | **6.462** |
|  |  | **Medication costs** | **2.014** | **1.767** | **2.686** | **1.726** | **3.770** |
|  |  | **Laboratory analysis** | **3** | **2** | **3** | **2** | **4** |
|  |  | **Eye worm removal** | **72** | **58** | **86** | **44** | **100** |
|  |  | **Sum** | **6.753** | **5.576** | **8.352** | **4.612** | **10.336** |
|  | **Non health-care costs** | **Transport** | **2.892** | **2.536** | **3.855** | **2.479** | **6.148** |
|  |  | **Food** | **500** | **262** | **737** | **250** | **750** |
|  |  | **Sum** | **3.391** | **2.798** | **4.592** | **2.729** | **6.898** |
| **Direct costs total** |  |  | **10.144** | **8.374** | **12.944** | **7.342** | **17.235** |
|  |  |  |  |  |  |  |  |
| **Indirect costs** | **Loss of income** | **Sicke leave in days** | **24.599** | **4.090** | **47.494** | **2.242** | **59.472** |
|  |  |  |  |  |  |  |  |
| **Total costs** |  |  | **34.744** | **12.464** | **60.438** | **9.584** | **76.707** |
| **Per 100.000 rural population** | | | | | | | |
| **Cost Type** | **Item** | **Components** | **Estimated median costs** | **95% CI lower** | **95% CI upper** | **Min** | **Max** |
| **Direct costs** | **Health care costs** | **Consultation** | **126.305.807** | **101.530.616** | **151.056.081** | **76.938.233** | **175.000.919** |
|  |  | **Medication costs** | **54.557.689** | **47.848.238** | **72.731.488** | **46.745.975** | **102.114.518** |
|  |  | **Laboratory analysis** | **67.707** | **54.436** | **80.977** | **42.249** | **95.602** |
|  |  | **Eye worm removal** | **1.949.922** | **1.567.543** | **2.331.815** | **1.191.365** | **2.719.638** |
|  |  | **Sum** | **182.881.125** | **151.000.833** | **226.200.361** | **124.917.822** | **279.930.676** |
|  | **Non health-care costs** | **Transport** | **78.309.427** | **68.679.114** | **104.397.313** | **67.140.821** | **166.514.576** |
|  |  | **Food** | **13.541.045** | **7.108.649** | **19.972.086** | **6.770.658** | **20.311.161** |
|  |  | **Sum** | **91.850.471** | **75.787.763** | **124.369.399** | **73.911.478** | **186.825.737** |
| **Direct costs total** |  |  | **274.731.596** | **226.788.596** | **350.569.760** | **198.829.300** | **466.756.412** |
|  |  |  |  |  |  |  |  |
| **Indirect costs** | **Loss of income** | **Sicke leave in days** | **666.214.030** | **110.774.189** | **1.286.259.216** | **60.719.529** | **1.610.662.280** |
|  |  |  |  |  |  |  |  |
| **Total costs** |  |  | **940.945.626** | **337.562.785** | **1.636.828.976** | **259.548.829** | **2.077.418.692** |
| **For the rural population of Gabon (n=204 474)** | | | | | | | |
| **Cost Type** | **Item** | **Components** | **Estimated median costs** | **95% CI lower** | **95% CI upper** | **Min** | **Max** |
| **Direct costs** | **Health care costs** | **Consultation** | **258.262.535** | **207.603.712** | **308.870.412** | **4.090.996.077.652** | **2.083.689.763.898** |
|  |  | **Medication costs** | **111.556.289** | **97.837.206** | **148.716.983** | **1.969.760.041.304** | **1.266.003.976.591** |
|  |  | **Laboratory analysis** | **138.442** | **111.308** | **165.577** | **2.193.071.979** | **1.144.211.468** |
|  |  | **Eye worm removal** | **3.987.084** | **3.205.217** | **4.767.954** | **63.151.671.379** | **32.265.296.446** |
|  |  | **Sum** | **373.944.351** | **308.757.443** | **462.520.927** | **6.126.100.862.315** | **3.383.103.248.402** |
|  | **Non health-care costs** | **Transport** | **160.122.418** | **140.430.932** | **213.465.361** | **2.827.353.870.886** | **1.818.350.058.460** |
|  |  | **Food** | **27.687.915** | **14.535.339** | **40.837.723** | **540.896.632.506** | **183.367.222.356** |
|  |  | **Sum** | **187.810.333** | **154.966.271** | **254.303.084** | **3.368.250.503.391** | **2.001.717.280.816** |
| **Direct costs total** |  |  | **561.754.684** | **463.723.714** | **716.824.011** | **9.494.351.365.706** | **5.384.820.529.219** |
|  |  |  |  |  |  |  |  |
| **Indirect costs** | **Loss of income** | **Sicke leave in days** | **1.362.234.476** | **226.504.415** | **2.630.065.669** | **34.835.283.418.044** | **1.644.444.584.778** |
|  |  |  |  |  |  |  |  |
| **Total costs** |  |  | **1.923.989.159** | **690.228.129** | **3.346.889.680** | **44.329.634.783.750** | **7.029.265.113.996** |

**S1_Table_G: Cost estimates in USD.**

| **Median costs per loiasis positive individual** | | | | | | | |
| --- | --- | --- | --- | --- | --- | --- | --- |
| **Cost Type** | **Item** | **Components** | **Estimated median costs** | **95% CI lower** | **95% CI upper** | **Min** | **Max** |
| **Direct costs** | **Health care costs** | **Consultation** | **8** | **6** | **9** | **5** | **11** |
|  |  | **Medication costs** | **3** | **3** | **4** | **3** | **6** |
|  |  | **Laboratory analysis** | **0,004** | **0,003** | **0,005** | **0,003** | **0,006** |
|  |  | **Eye worm removal** | **0,120** | **0,096** | **0,143** | **0,073** | **0,167** |
|  |  | **Sum** | **11** | **9** | **14** | **8** | **17** |
|  | **Non health-care costs** | **Transport** | **5** | **4** | **6** | **4** | **10** |
|  |  | **Food** | **1** | **0,437** | **1** | **0** | **1** |
|  |  | **Sum** | **6** | **5** | **8** | **5** | **11** |
| **Direct costs total** |  |  | **17** | **14** | **22** | **12** | **29** |
|  |  |  |  |  |  |  |  |
| **Indirect costs** | **Loss of income** | **Sicke leave in days** | **41** | **7** | **79** | **4** | **99** |
|  |  |  |  |  |  |  |  |
| **Total costs** |  |  | **58** | **21** | **101** | **16** | **128** |
| **Per 100.000 rural population** | | | | | | | |
| **Cost Type** | **Item** | **Components** | **Estimated median costs** | **95% CI lower** | **95% CI upper** | **Min** | **Max** |
| **Direct costs** | **Health care costs** | **Consultation** | **210.461** | **169.178** | **251.701** | **128.200** | **291.600** |
|  |  | **Medication costs** | **90.908** | **79.728** | **121.191** | **77.892** | **170.151** |
|  |  | **Laboratory analysis** | **113** | **91** | **135** | **70** | **159** |
|  |  | **Eye worm removal** | **3.249** | **2.612** | **3.885** | **1.985** | **4.532** |
|  |  | **Sum** | **304.731** | **251.609** | **376.913** | **208.148** | **466.442** |
|  | **Non health-care costs** | **Transport** | **130.485** | **114.438** | **173.955** | **111.875** | **277.460** |
|  |  | **Food** | **22.563** | **11.845** | **33.279** | **11.282** | **33.844** |
|  |  | **Sum** | **153.048** | **126.283** | **207.234** | **123.157** | **311.304** |
| **Direct costs total** |  |  | **457.779** | **377.893** | **584.147** | **331.305** | **777.746** |
|  |  |  |  |  |  |  |  |
| **Indirect costs** | **Loss of income** | **Sicke leave in days** | **1.110.098** | **184.581** | **2.143.265** | **101.176** | **2.683.811** |
|  |  |  |  |  |  |  |  |
| **Total costs** |  |  | **1.567.877** | **562.473** | **2.727.412** | **432.480** | **3.461.557** |
| **For the rural population of Gabon (n=204 474)** | | | | | | | |
| **Cost Type** | **Item** | **Components** | **Estimated median costs** | **95% CI lower** | **95% CI upper** | **Min** | **Max** |
| **Direct costs** | **Health care costs** | **Consultation** | **430.337** | **345.925** | **514.664** | **6.816.736.224** | **3.472.006.138** |
|  |  | **Medication costs** | **185.884** | **163.024** | **247.804** | **3.282.167.563** | **2.109.514.408** |
|  |  | **Laboratory analysis** | **231** | **185** | **276** | **3.654.267** | **1.906.574** |
|  |  | **Eye worm removal** | **6.644** | **5.341** | **7.945** | **105.228.232** | **53.762.949** |
|  |  | **Sum** | **623.095** | **514.476** | **770.688** | **10.207.786.287** | **5.637.190.070** |
|  | **Non health-care costs** | **Transport** | **266.808** | **233.997** | **355.693** | **4.711.157.181** | **3.029.876.460** |
|  |  | **Food** | **46.136** | **24.220** | **68.047** | **901.284.088** | **305.540.744** |
|  |  | **Sum** | **312.944** | **258.217** | **423.740** | **5.612.441.269** | **3.335.417.204** |
| **Direct costs total** |  |  | **936.039** | **772.693** | **1.194.428** | **15.820.227.556** | **8.972.607.274** |
|  |  |  |  |  |  |  |  |
| **Indirect costs** | **Loss of income** | **Sicke leave in days** | **2.269.861** | **377.419** | **4.382.420** | **58.045.261.802** | **2.740.101.618** |
|  |  |  |  |  |  |  |  |
| **Total costs** |  |  | **3.205.901** | **1.150.112** | **5.576.848** | **73.865.489.359** | **11.712.708.891** |
